# Exceptionally high but still growing predatory reef fish biomass after 23 years of protection in a Marine Protected Area

**DOI:** 10.1371/journal.pone.0246335

**Published:** 2021-02-08

**Authors:** Irene Rojo, José Daniel Anadón, José Antonio García-Charton

**Affiliations:** 1 Departamento de Ecología e Hidrología, Universidad de Murcia, Murcia, Spain; 2 Instituto Pirenaico de Ecología, Consejo Superior de Investigaciones Científicas, Zaragoza, Spain; University of Sydney, AUSTRALIA

## Abstract

Marine Protected Areas (MPAs) help replenish fish assemblages, though different trophic levels may show diverse recovery patterns. Long-term protection is required to achieve total recovery but poaching events may prevent the achievement of full carrying capacity. Here, we have analysed the effect of long-term protection on the entire reef fish community and the different trophic levels in the Cabo de Palos-Islas Hormigas MPA (SE Spain; SW Mediterranean Sea) in order to assess their recovery patterns after 23 years of protection. We compared the values for carrying capacity obtained with the maximum values achieved at regional scale, and we assessed the effect of a reduction in the surveillance over a few years, during which poaching events increased, on the recovery patterns. We found that, overall, biomass of fishes increased with time while density diminished. In particular, piscivorous and macro-invertivore fish increased while the other trophic groups remained constant or declined, suggesting top-down processes. For the entire study period, those trophic groups were approaching carrying capacity; however, when accounting only for the period in which enforcement was high and constant, they grew exponentially, indicating that full carrying capacity may have not been achieved yet. When compared to other Mediterranean MPAs, the Cabo de Palos-Islas Hormigas MPA showed values for biomass that were disproportionately higher, suggesting that local factors, such as habitat structure and associated oceanographic processes, may be responsible for the dynamics found. Our results help to understand the potential trajectories of fish assemblages over a consolidated MPA and highlight empirically how the reduction of surveillance in a period may change the recovery patterns.

## 1. Introduction

Marine Protected Areas (hereafter MPAs) are spatial management tools for fisheries regulation and biodiversity conservation [1]. By banning and/or limiting the fisheries activity, they allow the recovery of the species inside their limits, both in terms of biomass and abundance [2, 3]. However, protection does not appear to benefit all species in the same way. The general theory across many terrestrial and aquatic ecosystems states that increasing total biomass in a system leads to a change from top heavy to bottom heavy pyramids (i.e. more prey biomass per predator biomass; [4]). However, in coastal ecosystems, and particularly in MPAs, mature systems are expected to show inverse trophic pyramids [5, 6], with around 50% of fish biomass represented by high level predators [7, 8], which are expected to benefit the most from protection measures (e.g. [2, 3, 9–12]). As a consequence, cascading effects are expected to occur on prey and basal species [13, 14], following top-down (or consumer-control) dynamics (i.e. predators controlling the abundance of preys; [15, 16]). These top-down forces cannot easily be identified outside MPAs because fishing pressure has excessively targeted high level predators [17, 18] and currently most predator species are virtually absent from coastal ecosystems [19, 20].

A particular issue is whether the increase in fish populations due to the effects of protection is greater in terms of numerical abundance (i.e. density) or biomass for the different species and trophic groups, given the great variety of body size and schooling behaviour among them [21]. The patterns shown by abundance and biomass data are expected to be different, the increase in biomass being generally greater than that of abundance [22], especially where ecological gradients are short (i.e. environmental gradients covering a small portion of the whole range of the species) [23], when the fish community sampled includes a wide variety of guilds, or when large and/or small organisms are undersampled [24]. This is particularly evident in the situations where a loss of large body sized high level predators occurs, due to a shift from dominance by a few large high level predators to numerous much smaller, lower trophic level consumers [6].

Long-term protection is necessary to achieve complete recovery of the species. There are several examples where time since the beginning of protection appears as an important factor driving the ecological effectiveness of MPAs [2, 3, 11, 13, 25], with species reaching carrying capacity after 20 years of protection [26], and even increasing in a non-saturating way after more than 20 years [5, 27]. However, long-term protection is more likely to suffer from budget cuts at any time and from increases in poaching events [28]. Hence, high enforcement (i.e. poaching very occasional if any, and patrolling very active and continuous, *sensu* Guidetti et al. [10]) is a crucial condition for the correct functioning of MPAs in terms of recovery of the species [3, 10, 29], and has even been found as a critical factor determining the effectiveness of MPAs worldwide [30, 31].

Most of the studies assessing the effectiveness of MPAs are control-impact studies (i.e. spatial comparison of abundance, biomass or diversity between MPAs and adjacent unprotected areas), as a ’space-for-time’ alternative to long-term studies [7], due to the difficulty of achieving continuous data for long periods in marine studies. However, this type of study does not allow the assessment of ecological resilience, nor does it enable a full understanding of community changes through time, while continuous time series do [32]. Therefore, any long-term study aiming at ascertaining the extent of recovery of fish biomass and/or abundance within MPAs after the cessation of fishing activities should establish how near or far the values found are from the local and regional carrying capacity [26, 33, 34]. By carrying capacity of the environment we understand the abundance and biomass of the species or the trophic groups being studied at which the growth rate is zero [27], which will be attained within a period of time. The carrying capacity varies with factors such as habitat structure, food availability, primary productivity and climatic conditions [35]. However, in situations in which these variables remain constant in the area, the local carrying capacity can be calculated from the long-term data on the fish communities or the trophic groups [26, 27, 33].

In this study we analysed the long-term effect of protection on both the density and biomass of the reef fish community in the Cabo de Palos-Islas Hormigas MPA (SE Spain), which had been protected over 23 years at the time of the research. Specifically, the aims of the study were to assess the recovery patterns and to ascertain whether fish biomass and density of (i) the whole fish community and (ii) the different trophic groups, have reached the carrying capacity of the system, and in that case, to obtain the value attained (considering the whole period of study); furthermore, (iii) we discuss observed temporal response in the context of current knowledge on fish assemblages in other Mediterranean areas. In addition, because at a certain time surveillance abruptly decreased and poaching events increased in frequency in the MPA for a few years, (iv) we empirically assessed the shape of the recovery trajectories for the whole fish community and fish trophic groups under changing enforcement levels. Based on the previous literature, we hypothesize that the biomass of the whole fish community will show greater increases than the density for the whole period of study; high-level trophic groups will show greater responses in terms of both density and biomass and will prompt top-down control over the other trophic groups. Moreover, we will expect recovery trajectories to reach higher values for the first period of time, during which the enforcement was high and continuous, than for the whole period of study.

## 2. Material and methods

### 2.1. Study area and sampling methodology

**Fig 1:**
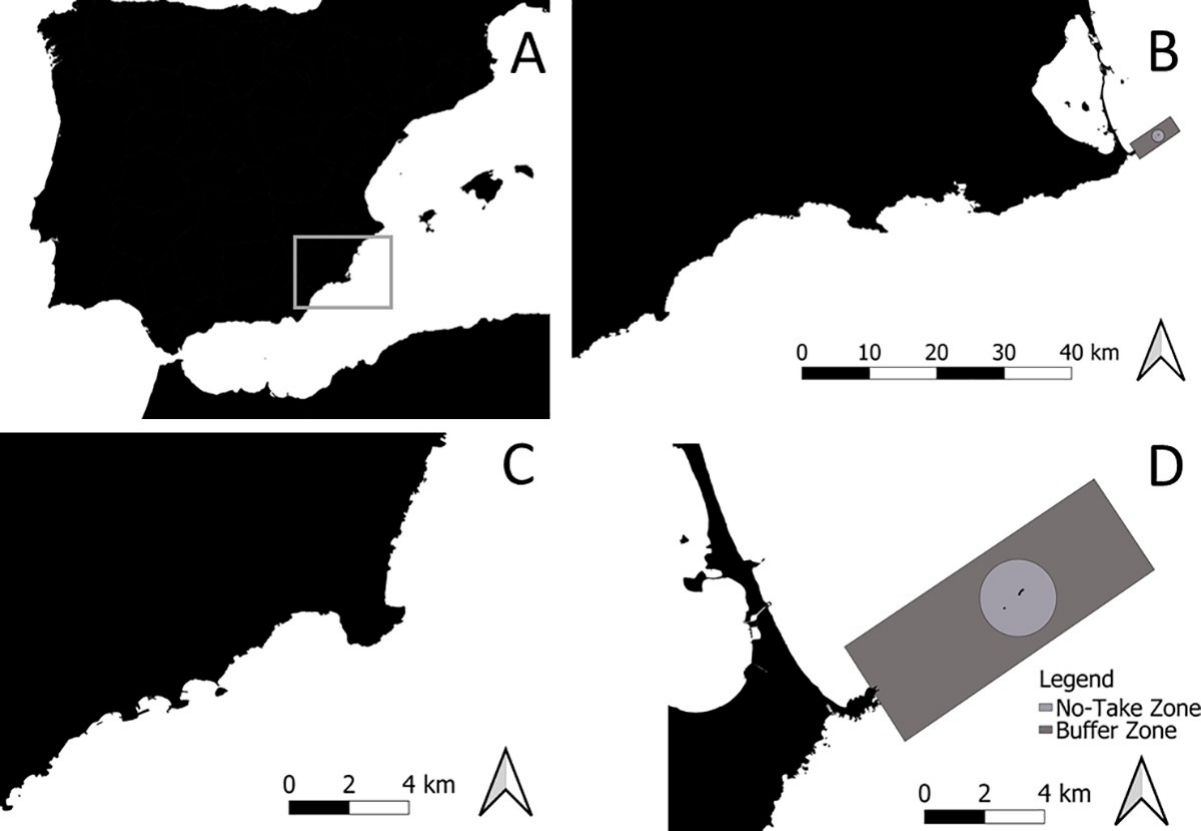
Study area. Study area showing the Cabo de Palos-Islas Hormigas MPA and the Cabo Cope unprotected area and their location in the SE of Spain, indicating the protection levels of the MPA.

The present study was carried out in the Cabo de Palos-Islas Hormigas marine reserve (hereafter Cabo de Palos) and the area surrounding Cabo Cope, which is unprotected and has been used as a control, both areas located on the coast of Murcia (SE Spain, SW Mediterranean Sea; Fig 1). The Cabo de Palos MPA was created in 1995 under fisheries legislation, and consists of a no-take zone occupying 270 ha, where all activities are banned, and a partially protected area surrounding the no-take zone and encompassing 1661 ha, where recreational diving and artisanal fisheries are allowed but strongly regulated (Fig 1). The habitat in this MPA consists of a series of sea mountains and the small archipelago of Hormigas islands (where the no-take zone is located), which are aligned towards the open sea in a northeast direction as a continuation of the mountain range that ends in the peninsula of the cape of Palos. The sea mountains extend from 3 to 10 m depth down to 30 to 60 m. In the shallower areas (<16 m deep) there is a heterogeneous combination of rocky reefs, patches of sand, and meadows of the seagrass *Posidonia oceanica* that adopt diverse morphological configurations. The infralittoral zone is covered by photophylic algae interspersed with precoralligenous biocoenoses (in the areas more protected from light), and the circalittoral zone is dominated by coralligenous habitats formed mainly by the gorgonians *Paramuricea clavata* and *Eunicella singularis*, extending down to the deepest areas occupied by detritic biocoenoses [36].

Cabo Cope is an unprotected area located in the southernmost sector of the coast of Murcia, at about 70 km from Cabo de Palos (Fig 1), which has been taken as a control area in the present study. The selection of this area was based on its relative similarity in terms of habitat (as compared to other rocky reefs in the region, given the singularity of the habitat configuration in the MPA). This coastal area consists of a high cliff interspersed with small sandy beaches, in which several rocky formations stand out, such as the ’Fraile’ island and the cape of Cope, a limestone promontory plunging into the sea and extending for more than 3 km. The sea bottom consists in rocky boulders, sand and patches of seagrass meadows in the shallower areas, with detrital habitats at greater depths [36].

In the Cabo de Palos MPA, a monitoring program of a variety of indicators of MPA performance (including fish assemblages) has been in place since 1996 on an annual basis and in the summer months, between the end of June and the end of September, in order to keep the environmental variables as constant as possible; the present study includes data up to 2018 (except for the years 1999, 2001, 2011 and 2012 for various reasons including budget cuts). Although the entire MPA is surveyed every year to visually assess fish assemblages (see below), only the data issued from the sea mountains (in the partially protected area) and the Hormigas islands (the no-take zone) were included in this study, thus excluding the data taken in the coastal area, because this part is very different, both in terms of habitat structure and human uses: recreational fishing from the coast is allowed, and the area is very popular with bathers, snorkellers and other nautical activities, mainly in summer. Each of the sea mountains and islets were considered a sampling site, and in each site, one to three transects were performed (see data at http://hdl.handle.net/10201/92641 for details on the sites and replicates sampled each year).

At Cabo Cope, the monitoring was performed from 1996 to 2018, but the data series is not continuous. For density, there are available data for 1996, 2000, 2002 to 2006, 2009 and 2016 to 2018. For biomass, though, data are available for the same years except 1996 and 2000. Seven to nine sites were selected each year, and three transects were performed at each site (see data at http://hdl.handle.net/10201/92641 for details on the sites sampled each year).

The monitoring was performed through underwater visual censuses (UVC) using belt transects of 50×5 m^2^. With this methodology, all fish encountered in the sampled area were recorded, except small-sized, cryptic species that live associated with the bottom [37]. For each observation the name of the species was noted and the number of individuals was assigned to one of nine predetermined abundance classes [38]. The size of each individual sighted was visually estimated by considering 2-cm length classes, except for larger fish species, for which 5-cm length classes were considered. All UVCs were performed on rocky substrates at depths between 16 and 20 m. UVCs were carried out between 10 and 15 h, when light and water conditions were optimal (see García-Charton [21] for further details on the UVC sampling methodology used). In general, enforcement level (*sensu* Guidetti et al. [10]) can be considered as being high during most of the sampling period. However, between the years 2010 and 2012 a drastic reduction of the surveillance occurred due to economic constraints, and from 2013 surveillance slowly started to be restored until 2014. Consequently, there were numerous events of poaching during that period. As a result of the limited surveillance, there are no official data on illegal fishing reports, but considerable indirect evidence was recorded, such as harmed individuals, harpoons found within the MPA, photos of catches in recreational fishing forums of fish that attain very large sizes which are not found elsewhere in the area, recurrent complaints by artisanal fishermen and reports made by the local police in restaurants in the area.

### 2.2. Data analysis

#### 2.2.1. Growth curves and model selection

We selected seven ecologically meaningful population models (Table 1) to analyse the recovery trajectories and rates of change of each descriptor measured in the Cabo de Palos MPA over the 23 years of study (see below). Both linear and exponential models show continuous growth, for which it is assumed that there are surplus resources available for the populations throughout the period studied. The von Bertalanffy, asymptotic, logistic and Gompertz are all models that approach a carrying capacity at diverse growth rates. Specifically, the logistic and Gompertz are sigmoidal curves, but the logistic is symmetrical while the Gompertz is asymmetrical, thus the asymptotic value is approached more gradually. The Ricker model assumes that there is a peak in the resource, which limits the growth of the populations and forces them to slow down.

**Table 1:** Growth population equations used to model temporal trends in fish responses.

Model	Equation	Ecological meaning
Linear	y = a+m·time	Constant rates of increase or decline (m) of the populations from an initial density or biomass (a)
Exponential	y = a·e^b·time^	Populations increase or decrease at an exponential rate (b) from an initial density or biomass (a), and the rate is independent of populations size
von Bertalanffy	y = K(1−e^−r(time−t0)^)	A rapid increase (*r*) that slows down as the population reaches the carrying capacity (*K*), where t0 is the theoretical time when *y* = 0
Logistic	$y=\frac{K}{1+(\frac{K-N0}{N0})e^{-r\cdot time}}$	After an initial exponential rate, growth rate (r) declines as the population reaches a carrying capacity (K), where N0 is the initial value for the dependent variable
Asymptotic	y = K+(N0−K)e^−r·time^	The population reaches a carrying capacity (K) at a constant rate (r), where N0 is the initial value for the dependent variable
Gompertz	$y=K\cdot e^{{(b\cdot e}^{\left(r\cdot time\right)})}$	Growth is slowest at the beginning and at the end of the period (b; r), and the population approaches the carrying capacity (K) gradually
Ricker	y = N0+(a·time)e^−b·time^	The population reaches a maximum peak by an initial rate of increase (a) and decline (b)

These population models were fitted through non-linear curves, applying the nls() function from the stats package [39]. Model selection was based on the AICc (the Akaike Information Criterion corrected for small sample sizes; [40]). Models with the lowest values of AICc were considered to have better support of the data. When Δ AICc between models was ≤ 2 we considered them to be equivalent in support [41]. Thus, we additionally calculated the proportion of variation explained by the models using a nonlinear approximation for *R*^*2*^ (following McClanahan et al. [5]):

$$R^{2}=1-\frac{{SS}_{reg}}{{SS}_{tot}},$$

where SS_reg_ is the residual sum of squares given the model, and SS_tot_ is the total sum of squares in the response. When Δ AICc ≤ 2, for printing purposes we selected the model with the greatest *R*^2^. All analyses were performed in R [39].

#### 2.2.2. Descriptors assessed

We carried out separate analyses for the density (abundance · 250 m^-2^) and biomass (expressed in g · 250 m^-2^) response variables. The latter was estimated through adequate length-weight relationships from local studies when available [42] and Fishbase [43] (see S1 Table for detailed information on the length-weight conversion and the *a* and *b* coefficients for each species). For each response variable, we used total values (considering all species), reduced values (excluding pelagic species) and trophic groups as descriptors, gathered from Bell and Harmelin-Vivien [44]. As trophic groups, we included piscivorous fishes (species mainly feeding on fish, but also on cephalopods and macroinvertebrates, and scavenger species), piscivorous reduced (same as before excluding the pelagic species, which may distort the results because they may form very large shoals aggregated in certain parts of the sea mountains and islands), macro-invertivores (species with diets based on medium-sized invertebrates and, to a lesser extent, on small fishes), micro-invertivores (species feeding on small invertebrates), omnivorous fishes (species feeding over various trophic levels), herbivorous (species with diets strictly based on primary producers, such as seagrasses or macroalgae), planktivorous (species feeding on zoo- and phytoplankton) and detritivorous fishes (species feeding mainly on organic matter accumulated in the sediment) (Table 2).

**Table 2:** List of the species found in the study and their trophic position and pelagic mobility.

Family	Species	Trophic group	Pelagic
Myliobatidae	*Myliobatis aquila*	MACRO	
Clupeidae	Sarda sarda*	PISC	X
Engraulidae	*Engraulis encrasicolus*	PLAN	X
Muraenidae	*Muraena helena*	PISC	
Belonidae	Belone belone*	PISC	X
Phycidae	*Phycis phycis*	PISC	
Serranidae	*Anthias anthias*	PLAN	X
	*Epinephelus costae*	PISC	
	*Epinephelus marginatus*	PISC	
	Epinephelus caninus*	PISC	
	*Mycteroperca rubra*	PISC	
	*Serranus atricauda*	MACRO	
	*Serranus cabrilla*	MACRO	
	*Serranus scriba*	MACRO	
Moronidae	*Dicentrarchus labrax*	PISC	
Apogonidae	*Apogon imberbis*	MICRO	
Carangidae	*Seriola dumerili*	PISC	X
	*Trachurus spp*.*	PLAN	X
	Pseudocaranx dentex*	PLAN	X
Scombridae	*Sarpa salpa*	HERB	
	*Euthynnus aletteratus*	PISC	X
Coryphaenidae	Coryphaena hippurus*	PISC	X
Haemulidae	*Pomadasys incisus*	MACRO	
	*Parapristipoma octolineatum*	MACRO	
Sciaenidae	*Sciaena umbra*	PISC	
Mullidae	*Mullus surmuletus*	DETR	
Sparidae	*Boops boops*	PLAN	X
	*Dentex dentex*	PISC	
	*Diplodus annularis*	OMNI	
	*Diplodus cervinus*	OMNI	
	*Diplodus puntazzo*	OMNI	
	*Diplodus sargus*	OMNI	
	*Diplodus vulgaris*	OMNI	
	*Oblada melanura*	PLAN	X
	*Pagrus pagrus*	OMNI	
	Pagrus auriga*	OMNI	
	*Sardina pilchardus*	PLAN	X
	*Sparus aurata*	OMNI	
	*Spondyliosoma cantharus*	OMNI	
	*Spicara smaris*	PLAN	X
	*Spicara maena*	PLAN	X
Pomacentridae	*Chromis chromis*	PLAN	X
Labridae	*Coris julis*	MICRO	
	*Labrus merula*	MICRO	
	*Labrus viridis*	MICRO	
	*Symphodus dordeleini*	MICRO	
	*Symphodus mediterraneus*	MICRO	
	*Symphodus melanocercus*	MICRO	
	*Symphodus ocellatus*	MICRO	
	*Symphodus roissali*	MICRO	
	*Symphodus cinereus*	MICRO	
	*Symphodus rostratus*	MICRO	
	*Symphodus tinca*	MICRO	
	*Thalasoma pavo*	MICRO	
Sphyraenidae	*Sphyraena viridensis*	PISC	X
Mugilidae	*Mugilidae spp*.	DETR	X
Scorpaenidae	Scorpaena maderensis*	MACRO	
	*Scorpaena scrofa*	PISC	
	*Scorpaena porcus*	MACRO	
	*Scorpaena notata*	MACRO	
Atherinidae	*Atherina sp*.*	PLAN	X
Molidae	Mola mola*	PLAN	X

PISC: piscivorous fishes; MACRO: macro-invertivores; MICRO: micro-invertivores; OMNI: omnivorous species; HERB: herbivorous species; PLAN: planktivorous fishes; DETR: detritivorous fishes. The asterisk indicates the species that have not been found in the Cabo Cope unprotected area.

We did not distinguish between levels of protection within the MPA, as previous studies in the area have found that even sedentary species exhibit movements between sea mountains and islands [45]. Moreover, the only fishing pressure in the partially protected area comes from small-scale fishing boats which very rarely operate in the marine reserve, and in general fish densities are not significantly different between the two protected levels [46]. Thus, we considered all sea mountains and islands to be representative of the habitat and the protected status regardless of the protection level of each one.

Considering the above-mentioned increase in poaching activity that occurred in the study area during the period 2010–2014, we carried out three separate analyses: an analysis for the whole studied period (1996–2018), an analysis that included only the period before the surveillance was interrupted and poaching events started to occur (1996–2009), and an analysis for the period in which the surveillance was fully restored after the interruption (2015–2018). This third time series is only four years long, and thus very limited in its ability for fitting temporal patterns to a model; however, we have included it here since it is still meaningful for indicating the temporal recovery trend once the surveillance measures were restored. Thus, for each descriptor (total, total reduced and each trophic group) we calculated a global yearly mean and standard error and performed the analyses of biomass and density, considering separately the whole period, the before-interruption and the after-restoration of surveillance periods. In order to set a control against which to compare the effect of long-term protection in the Cabo de Palos MPA, we calculated a global yearly mean and standard error for the biomass and density in the Cabo Cope unprotected area and represented it together with the protected data and selected population models.

In addition, in order to compare the values for carrying capacity obtained in the studied MPA in relation to the values for other MPAs at regional scale, we extracted the values for carrying capacity for the biomass of the whole fish assemblage and of the piscivorous group from studies developed in western Mediterranean MPAs. Values were obtained from the published figures through the GetData Graph Digitizer software, (available at http://getdata-graph-digitizer.com).

## 3. Results

### 3.1. Visually censused reef fish assemblage

In the 19 years surveyed over the 23 years of the study period (1996–2018), a total of 268,960 individuals belonging to 62 fish species in 23 families have been observed in the visually censused transects performed in the Cabo de Palos-Islas Hormigas MPA (Table 2). From these, 16 species (25.8%) were piscivorous, 9 species (14.5%) were macro-invertivores and 13 species (20.9%) were micro-invertivores. Omnivorous species represented the 14.5% of the fish assemblage (9 species), while 12 species (19.4%) were planktivorous, 2 (3.2%) species were detritivorous, and only one species (1.6%)—*Sarpa salpa—*has a strict herbivorous diet. Regarding their life habits, 19 species (30.6%) were pelagic, the rest being demersal species (Table 2). At Cabo Cope, a total of 172,473 individuals belonging to 52 species in 17 families have been observed along the transects in the 11 years surveyed over the same period (Table 2). Of these, 12 species were piscivorous (23.1%), 8 species (15.4%) were macro-invertivores, 13 species (25%) were micro-invertivores, 8 species were omnivorous (15.4%), 8 species (15.4%) were planktivorous, 2 (3.8%) species were detritivorous, and again only one species (1.9%) was herbivorous (Table 2).

### 3.2. Model selection

Most selected models explaining the temporal change in fish biomass in the Cabo de Palos MPA for the whole period of study were logistic, while, when considering separately the first and last periods of study, exponential models were the ones that best fit the data. The selected models explained part of the variation in the data (total period *R*^*2*^ = 41.14% ± 7.63%; first period *R*^*2*^ = 53.18% ± 8.72%; last period *R*^*2*^ = 79.02% ± 7.64%; mean values across trophic levels ± SE). In the case of fish density, though, most data fitted Ricker models when accounting for the whole and last periods of study, and linear models for the first period. In this case, the variance explained by the models was: total period *R*^*2*^ = 49.10% ± 6.46%; first period *R*^*2*^ = 45.15% ± 8.55%; last period *R*^*2*^ = 60.31% ± 10.37% (mean ± SE).

### 3.2. General responses to long-term protection

**Fig 2:**
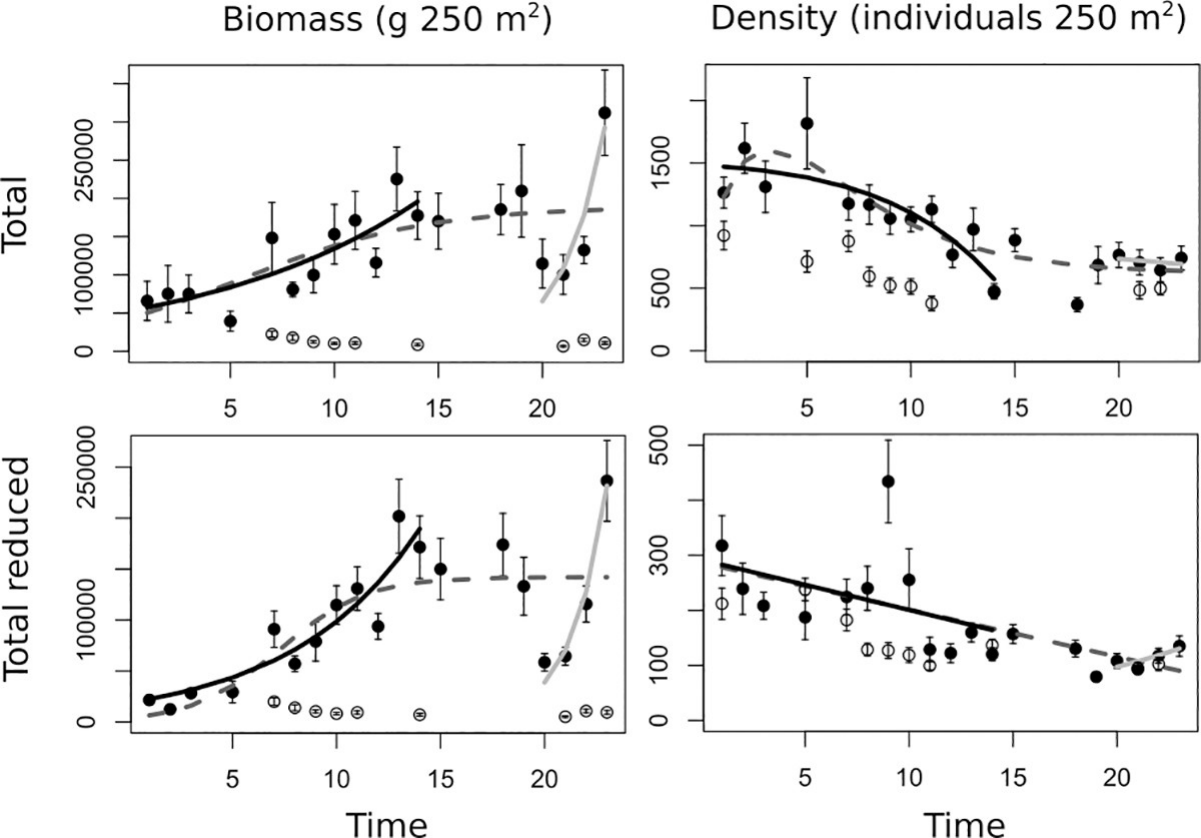
Population trajectories for the descriptors related to the whole fish community in the Cabo de Palos-Islas Hormigas MPA and in the Cabo Cope unprotected area. Population trajectories are shown for the biomass (expressed as g · 250 m^-2^) and density (number of individuals · 250 m^-2^) response variables (± SE) for the Cabo de Palos MPA (black circles) and the Cabo Cope unprotected area (white circles). The dashed line indicates the model selected when the whole period of study was considered. The black line indicates the population trajectory during the first period in which surveillance was high and constant. The light grey line indicates the trajectory of the fish after the reestablishment of the surveillance.

Growth models showed that, for the whole study period, the fish biomass increased dramatically with time, reaching a carrying capacity of 189,400 g 250 m^-2^ between 10 and 15 years after MPA inception (Fig 2, Table 3), while the opposite pattern was found for density, for which the variable appeared to decrease with time from 1,264 to 741 individuals 250 m^-2^ (Fig 2). These patterns were consistent both for the total and reduced descriptors (i.e. after extracting the contribution of pelagic species to total values), so that reduced biomass reached a carrying capacity of 142,200 g 250 m^-2^, and reduced density decreased with time from 317 to 134 individuals 250 m^-2^ (Fig 2, Table 3). In contrast, the values for biomass at Cabo Cope remained constant and at very low values compared to Cabo de Palos, around the 10,000 and 13,000 g 250 m^-2^ over the period of study for the total and reduced descriptors, respectively. In the case of density, values were more variable and more similar to the values for density reached in Cabo de Palos MPA (Fig 2).

**Table 3:** Carrying capacity values obtained for the biomass (g 250 m^-2^) and density (abundance 250 m^-2^) variables through the logistic models for the whole period of study and those calculated from the initial exponential trend during the first period.

Variable	Descriptor	Carrying capacity from the whole period of study	Carrying capacity from the initial exponential function
Biomass	Piscivores	16.28 · 10^4^	54.52 · 10^5^
Biomass	Piscivores reduced	12.20 · 10^4^	19.58 · 10^6^
Biomass	Total	18.94 · 10^4^	12.34 · 10^6^
Biomass	Total reduced	14.22 · 10^4^	36.09 · 10^6^
Density	Piscivores reduced	22.53	15.03 · 10^3^

### 3.3. Response of trophic groups to long-term protection

**Fig 3:**
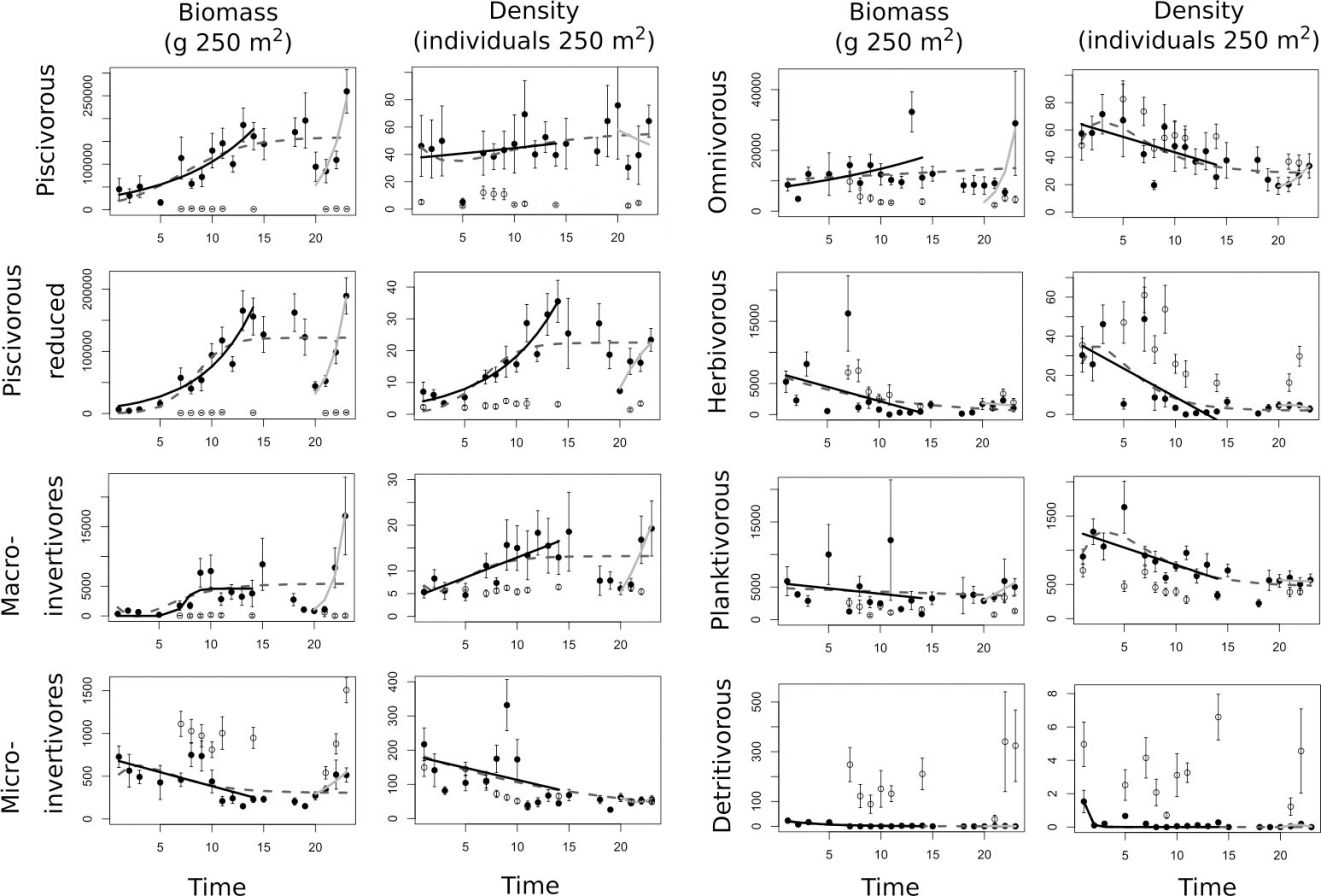
Population trajectories for the trophic descriptors in the Cabo de Palos-Islas Hormigas MPA and in the Cabo Cope unprotected area. Population trajectories are shown for the biomass (expressed as g 250 m^-2^) and density (individuals 250 m^-2^) response variables (± SE) for the Cabo de Palos MPA (black circles) and the Cabo Cope unprotected area (white circles). The dashed line indicates the model selected when the whole period of study was considered. The black line indicates the population trajectory during the first period in which surveillance was high and constant. The light grey line indicates the trajectory of the fish after the reestablishment of the surveillance.

Different patterns arose when looking at the temporal trajectory of the biomass of fish trophic groups throughout the whole period of study. Biomass of piscivorous and piscivorous reduced increased with time following logistic curves, thus reaching a carrying capacity of 162,800 and 122,200 g 250 m^-2^, respectively (Fig 3, Table 3). Omnivorous and macro-invertivores remained almost constant in the period of study at around 115,000 and 3,000 g 250 m^-2^, respectively, although the latter showed a strong increase in the last two years. Biomass of micro-invertivores, herbivorous, planktivorous and detritivorous fishes, however, exhibited a population decrease (Fig 3). At Cabo Cope, all trophic groups showed very low and constant biomass values over the study period, except micro-invertivores, herbivorous and detritivorous species, which exhibited higher values than at Cabo de Palos in most years (Fig 3).

**Fig 4:**
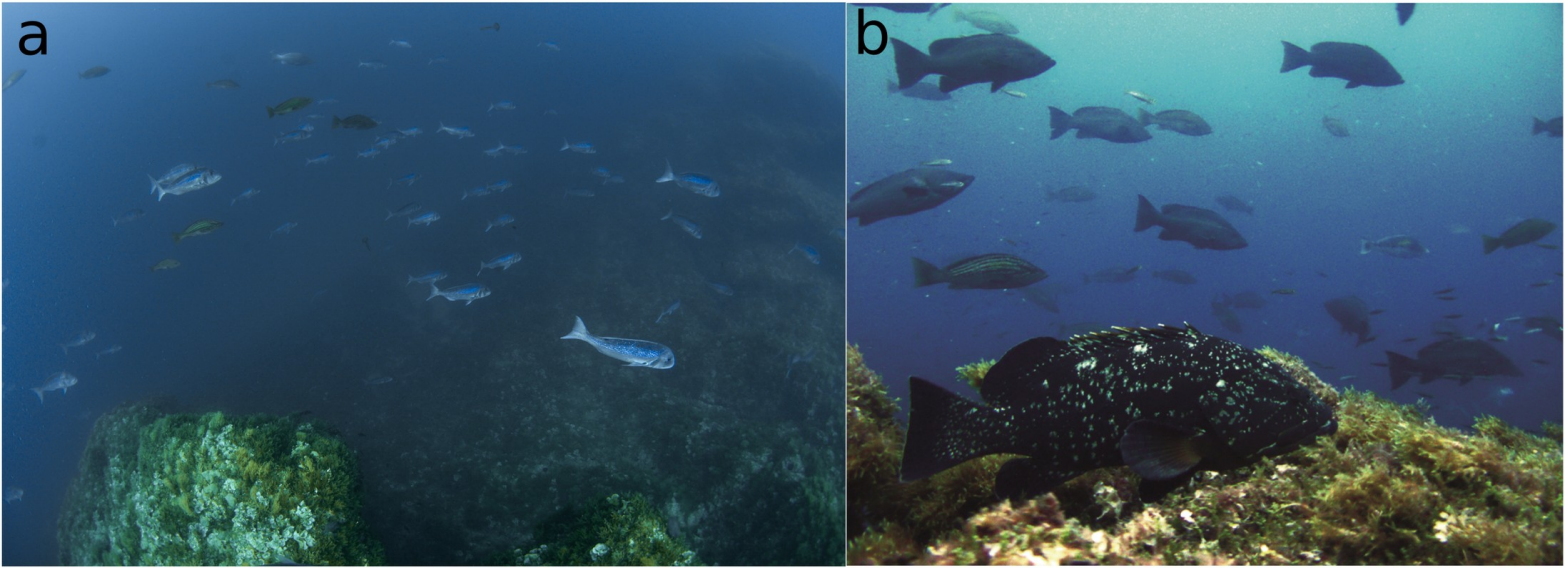
Abundance of predatory species in the Cabo de Palos-Islas Hormigas MPA. a) A large group of *Dentex dentex* interspersed with individuals of *Epinephelus costae*; photo: Javi Ferrer; b) Individuals of the species *Epinephelus costae*, *Epinephelus marginatus* and *Mycteroperca rubra*; photo: Johann Mourier.

In the case of fish density, all trophic groups analysed, except the piscivorous, piscivorous reduced and macro-invertivores, showed a decline in observed numbers (Figs 3 and 4). In the case of piscivorous fishes, their density remained manly constant over the study period. For their part, both piscivorous reduced and macro-invertivores showed a remarkable increase, with the first group reaching a carrying capacity of 22.53 individuals 250 m^-2^ for the whole study period (Fig 3, Table 3), while macro-invertivores attained an asymptote of about 12.5 individuals 250 m^-2^ after 11 years of protection (Fig 3). The fact that the density of piscivorous fishes did not follow the same pattern as piscivorous reduced highlights the variability that pelagic species, particularly *Sphyraena* spp., which is the most abundant species, add to the group (Fig 3). Similarly to biomass, at Cabo Cope, all trophic groups showed very low values for density compared to Cabo de Palos over the study period, except the omnivorous, herbivorous and detritivorous groups, which exhibited higher values than at Cabo de Palos in most years (Fig 3).

### 3.4. Regional comparison of fish biomass

The values of biomass carrying capacity estimated in this MPA for the whole study period were extremely high compared to the range of values available in the literature. Sala et al. [47] found that the maximum fish total biomass recorded in their dataset, issued from 13 MPAs and 17 unprotected areas across the Mediterranean (data that were reused by Guidetti et al. [12]), was 29,575 g 250 m^-2^ in Tavolara MPA. In this MPA, Sayhoun et al. [48] found that the total biomass reached on protected rocky banks, a similar habitat to our study, was 67,676 g 250 m^2^. Regarding the biomass of apex predators, which included pelagic species such as *Sarda sarda*, *Seriola dumerili* or *Sphyraena viridensis*, Sala et al. [45] found 59,025 g 250 m^-2^ in the same protected area. For their part, Coll et al. [33] found a maximum biomass of fish species vulnerable to commercial and recreational fishing (i.e. *Muraena helena*, *Diplodus* spp., *Spondilosoma cantharus*, *Epinephelus* spp., *Labrus* spp., *Sciaena umbra* and *Scorpaena* spp.), in the MPAs of Es Freus, Palma bay and Northern Menorca of 5,810 g 250 m^-2^. In addition, García-Rubies et al. [26] in Medes Islands found a maximum fish biomass of 1821 g 250 m^-2^ for the total of fish species vulnerable to fishing (including the species *E*. *marginatus*, *D*. *cervinus*, *D*. *labrax*, *D*. *dentex*, *S*. *aurata*, *S*. *umbra*). In our study, in the Cabo de Palos-Islas Hormigas MPA the carrying capacity for the total assemblage reached a remarkable 189,400 g 250 m^-2^ and for the total reduced group 142,200 g 250 m^-2^, while the carrying capacity of piscivorous species reached 162,800 g 250 m^-2^ and for piscivorous without pelagic species reached 122,000 g 250 m^2^ (Fig 4, Table 3).

### 3.5. Effect of reduced surveillance

During the 2010–2014 period, when surveillance intensity decreased, the values for density and biomass dropped noticeably (Figs 2 and 3). When we fit the growth models separately for the periods immediately before and after this time, for most descriptors that showed an increase throughout the whole period (density and/or biomass of the whole assemblage and trophic groups), whatever the model selected, growth was generally much higher during the first period of the study (when surveillance remained high), fitting either to linear or exponential functions (Figs 2 and 3). Because logistic functions imply an initial exponential increase, for those descriptors in which total periods fitted a logistic model and the first period fitted an exponential function we calculated new values of carrying capacity based on the exponential trend of the first period: overall, the estimated values were 1 to 3 orders of magnitude greater than those estimated by considering data for the whole study period (Table 3). In the same way, the last period (after the full restoration of the surveillance activities) showed in general exponential and linear increases for most of the descriptors studied (Figs 2 and 3). Although these last models were fitted to data issued from 4 years only, the recovery after the reestablishment of surveillance is remarkable, requiring much less time to reach values equivalent and even higher to what previously required more than a decade.

## 4. Discussion

Assessing the long-term effects of protection on reef fish communities is essential to understand the ecological mechanisms occurring in MPAs [32]. Moreover, this information is necessary to maintain effective protection over a minimum number of years in order to achieve full conservation benefits [29]. Here, we assessed the responses to protection of the reef fish community as a whole and of fish species grouped in trophic levels, both in terms of density and biomass, in an ecologically effective temperate MPA 23 years after its establishment, and compared the results on the basis of the response of fishes in an unprotected area. We additionally compared the values for carrying capacity obtained with the range of values in MPAs at regional scale and discussed which factors may be contributing to the different carrying capacities achieved in marine ecosystems. Finally, we explored whether a prolonged episode of poaching was able to affect the possibility of reaching or not such a carrying capacity. Our study offers new insights into the effects of variable surveillance on the fish assemblages within a MPA, and the information gathered will help to better design and manage MPAs to promote the conservation of reef fish communities.

### Reserve effect on the fish community

Our results show that, when accounting for all species together, fish biomass increased dramatically after the inception of the protection measures in the Cabo de Palos-Islas Hormigas MPA, while it remained constant in the Cabo Cope unprotected area. This pattern is likely to be primarily due to the existence of strong fishing pressure in Cabo de Palos prior to the declaration of this protected area [9], a situation corroborated by the testimony of artisanal fishermen operating in the area, who in addition report very low fishing catch at that time. Noticeably, the patterns shown by the density of the whole fish community differed from what was exhibited by its biomass, so that the former decreased while the latter increased throughout the study period. The values for density looked similar to those found at Cabo Cope, highlighting that protection measures have a different effect over the two response variables [29]. This is likely due to the fact that at Cabo Cope the absence of protection allowed a constant number of young individuals (i.e. with low sizes) because fishing targets the larger ones [49] and thus competition for space may not be occurring, while protection at Cabo de Palos promoted the establishment of fewer individuals residing in the area, but of larger size, due to the increased survival of individuals of certain species within the reserve (which therefore reach greater age and size), indicating that an adult community is well-established in the area [50–52]. In fact, density-dependent mechanisms have been identified at Cabo de Palos [53], especially for those species having high territoriality, such as groupers, which may be displacing young individuals to areas outside the reserve [54], while this pattern has not been found in unprotected areas of the region [53].

When focusing on the trophic levels, we found contrasting responses among different trophic groups. The total response described above is mainly driven by piscivorous, piscivorous reduced and, to a lesser extent, macro-invertivores, which show an increase in biomass over the period of study at Cabo de Palos, while the others remained constant or declined, depending on the response variable. Previous studies have found higher abundance of high trophic-level species compared to other trophic groups in MPAs [2, 3, 9–12, 55], as being traditionally the most exploited by both commercial and recreational fisheries [17, 49], thus being the most benefitted from protection measures. This pattern has been widely reported in the Mediterranean Sea (e.g. [2, 3]) and elsewhere [55, 56]. The fact that piscivores and macro-invertivores increased over the period of study while other trophic groups remained constant or declined suggests the occurrence of top-down processes [16]. In fact, at Cabo Cope, where the abundance and biomass of high trophic groups is very low, the abundance and biomass of low trophic groups is even higher than at Cabo de Palos. Other long-term studies found trends of increasing abundance/biomass of different trophic groups, such as herbivores and invertebrate eaters, while some families displayed differing trends, suggesting that control effected by predators may be species- and site-specific [5]. Top-down processes have been documented for predatory fish preying on invertebrates in the Mediterranean Sea [57, 58] and elsewhere [33, 59]. Moreover, indirect mechanisms such as behaviorally-mediated effects have been proposed, in which changes in the abundance of a species result in an alteration in the behaviour of a second species (i.e. risk effect) that finally influences a third species [60]. In fact, these mechanisms have been identified in the area. In the Cabo de Palos MPA, where the density of groupers is very high, Hackradt et al. [53] found a negative effect on the density of combers (mesopredators), which can be attributed to either direct predation, competition or a prey-release effect, while the pattern was not found in the unprotected area studied. Thus, in the Cabo de Palos MPA, where fishing is prohibited or strongly regulated, and the decline in abundance and biomass has occurred over several trophic groups, the most probable explanation is the top-down control by an increase in the high trophic groups, either directly or indirectly. It should be noted here that the pattern found, that of increase in biomass and in the top levels of the trophic web, reported also for other MPAs, is at odds with the general ecology observation-based theory that predicts a shift to heavy bottom pyramids with increasing biomass [4]. Overall, this mismatch highlights the uniqueness of the dynamics observed in the most productive coastal systems, where MPAs are usually located, and that inverted trophic pyramids, probably favored by the high renewal rate of primary producers, are the expected dynamics in marine ecosystems [6].

### Fish carrying capacity and poaching

MPAs promote the recovery of the communities that live within their limits, providing that they are protected for long enough [61], and that enforcement is high and constant through time [3, 10]. In our study we have calculated values for carrying capacity of 189,400 g 250 m^-2^ for the whole fish community or 162,800 g 250 m^-2^ for piscivores. We considered that the carrying capacity must be constant over the studied period because habitat and food availability have not varied. In addition, despite temperature data in the area showing summer peaks in recent years, there is not an increase in total temperatures (Ruiz, Navarro, Orenes & García-Charton, unpublished data), and the fact that fish data remain constant at Cabo Cope indicates that temperature is not affecting the carrying capacity.

When we compare the values for biomass carrying capacity estimated in this MPA with the range of values available in the literature [12, 26, 33, 47, 48], we find that the recorded biomass values at Cabo de Palos-Islas Hormigas are disproportionately high. This difference could be explained, in addition to a particularly intense reserve effect, by several non-exclusive hypotheses. Firstly, it has been found that there is a tendency for large-sized individuals to live at deeper areas as a consequence of ontogenetic movements aimed at benefit from decreasing metabolic rates at lower temperatures [62], and as a way to differentiate their habitat niche as they grow [45]. Our study was performed at depths between 16 and 20 m, while Sala et al. [45] sampled rocky habitats at 8–12 m; Coll et al. [33] performed UVCs at 3–15 m depth, and García-Rubies et al. [26] spanned their sampling units between 10 and 20 m depth; therefore, this methodological difference could partly contribute to explain the differences found. In addition, the habitat structure in the area may be partially responsible for the patterns found. The singular arrangement of rocky reefs in this MPA, formed by steep and pointed rocky shoals and islands, provide a spatial reference for pelagic fish species [63], which are attracted by the MPA and thus benefit from the ‘reserve effect’, particularly those that are resident all year long, such as big shoals of large-sized greater amberjack (*Seriola dumerili*) and common dentex (*D*. *dentex*), which are observed around the rocky mountains for much of the year, as a likely realization of the "spatial reference hypothesis" [63] to explain the concentration of schooling pelagic fish around sea mountains, islands or banks. The fact that the ’reserve effect’ on biomass is detectable for both the total (i.e. all species) and total reduced (i.e. excluding pelagic species) descriptors corroborates that pelagic species are an intrinsic part of the fish assemblage in the area, regardless of the protection measures. Noteworthy is that, biomass not only of piscivores but also of piscivores reduced show a ‘reserve effect’. The explanation for this is may be that *Sphyraena* spp., which is the most abundant pelagic piscivorous species in the area, is resident in the MPA and is commonly found at any season. Additionally, the rocky and complex habitats favor the development of diverse and abundant reef fish fauna, because they provide gaps, cracks and fissures where demersal fishes can find resources (namely refuge and food) [21]. Moreover, the contribution of energetic subsidies to the local food web may determine the trophic structure [64–66]. Very high abundances of forage species such as *Boops boops* and *Engraulis encrasicolus* are typical in the area, probably caused by the strong water currents and swirls determined by the steep topography of sea mountains and islands, which in turn would cause locally increased primary productivity and associated increment in zooplankton biomass; in addition, very high abundances of the damselfish *Chromis chromis* are observed in the area, which are important prey species for demersal predatory species such as groupers (*Epinephelus* spp., *Mycteroperca rubra*) and the sparid common dentex, as well as mesopredators such as combers (*Serranus* sp.), among other species (Rojo and García-Charton, *personal observation*). Another possible source of energetic subsidy to the protected rocky reefs is the movement pattern of highly mobile piscivorous fishes to forage outside the MPA [67]. In our case, large numbers of greater amberjack and common dentex, most likely from the marine reserve, are frequently caught with small-scale fishing gear outside the MPA [68], thus suggesting that these movements from the marine reserve to neighbouring unprotected sites do indeed occur.

In our study, the time required to achieve the carrying capacity of the whole community ranged from 10 to 15 years depending on whether or not pelagic species were included in the analysis. The question of how many years are expected to elapse until the scale of the results of protection measures reach their maximum is not trivial, since it influences the expectations raised among potential users of the MPA. However, it must be taken into account that the carrying capacities have been calculated considering the entire study period, including some years in which the abundance and biomass of predatory species decreased drastically as a result of poaching; thus, this estimate may be unrealistic and may not correspond to the maximum possible values for biomass that the marine reserve may hold. Even so, the time taken to reach these asymptotic values for reef fish population biomass is longer than that found in other Mediterranean MPAs, such as the Es Freus and Palma MPAs (5 years; [33]), but is comparable to MPAs located elsewhere (10–20 years; [5, 32, 69, 70]). However, when we fit the temporal trajectories separately for the periods of high enforcement before and after those years in which surveillance was reduced, we observe that during the first 14-year period the population growth was clearly exponential, showing no signs of approaching an asymptote (see also García-Charton et al. [71]); under these circumstances, the estimated carrying capacity would be 1 to 3 orders of magnitude greater than that calculated for the entire study period. Other MPAs have shown continuous increases in their populations after decadal times of protection; for instance, in the Port-Cros National Park, which was established in 1963 and has had effective enforcement since the beginning, dusky grouper (*Epinephelus marginatus*) populations are still increasing in terms of biomass after 55 years of protection [72]. Similarly, a study performed in 2 MPAs in the central Philippines showed that biomass of predatory fish after 9 and 18 years fitted exponential models, and the carrying capacity estimated from the data gave unrealistically high values [27]. However, in the absence of baselines, it is difficult to ascertain how unrealistic those measurements are. It is absolutely necessary to continue the long-term monitoring efforts in order to check how long the fish biomass can continue to grow in this marine reserve. These findings may indicate that carrying capacity has not been achieved in the area and that the values for carrying capacity found when fitting the data to the whole study period are a result of the reduced surveillance during the middle period.

### Fish biomass recovery after recuperating high enforcement level

The last 4-year period showed a rapid increase of many descriptors of the fish assemblage. Some kind of exploitation of the resources may lead to a fast increase in the growth rate of the assemblages [73–75]. In our study, the fact that after the surveillance was restored the whole community and the trophic groups experienced a rapid increase may be partially due to the exploitation undergone during the poaching events. However, since it occurred over a very short period of time, it is more likely to be due to a change in behaviour rather than the recruitment of new individuals, as the pattern is consistent for both biomass and density. Recent studies have shown how fishes are able to discern between scuba divers and spearfishing divers, and behaviourally respond to increased spearfishing pressure by being more shy and elusive [76]. Furthermore, it has been documented that increasing depth acts as a refuge from fishing as most fishing techniques focus on shallow waters [77, 78]. In this sense, it is possible that during the period in which poaching was high and surveillance was interrupted, fish behaviour changed and individuals moved to deeper rocky reefs, returning to their natural depth ranges once surveillance had returned to previous levels, and thus being detected again by the routine monitoring UVCs of the marine reserve.

In conclusion, our study, although focusing on one single Mediterranean MPA, offers important insights into the long-term effects of protection on reef fish assemblages and the effects of a decrease in surveillance over fish communities. Over 23 years of protection this MPA has exerted a strong positive influence on the biomass of the whole reef fish community, as well as the density and biomass of predatory and medium-sized invertebrate feeder species, with disproportionately high values when compared to other Mediterranean MPAs, probably due to the habitat structure and related oceanographic processes in this MPA. This effect has taken 10 to 15 years to reach a level close to the maximum observed to date. On the other hand, a likely occurrence of a top-down process may have reduced the abundance and biomass of lower trophic groups. High enforcement is essential to achieve the recovery of the populations and modifying enforcement levels can vary the recovery trajectories; the occurrence of continuous episodes of poaching for several years has probably prevented the protected area from reaching its carrying capacity, so it is not yet known how long the density and biomass of the populations benefitting most from the protection measures could continue to grow. The continued monitoring of this marine reserve in the coming years is more necessary than ever, in order to better understand the mechanisms underlying the exceptional response of the reef fish assemblage to protection measures. In addition, complementary studies such as functional [9, 79] and tropho-dynamic [80] modelling of the reserve effect, as well as diet studies through stable isotopes [81], would help to fully understand the changes that have occurred among the trophic groups as a result of protection, and to explain the exceptionally high biomass values observed over time.

## Supporting information

S1 Table Length-weight conversion formula to estimate weight of fish from their total lengths, and *a* and *b* coefficients for each studied species. (DOCX)
