# Supplementary material for: Exceptionally high but still growing predatory reef fish biomass after 23 years of protection in a Marine Protected Area
Source: PLoS One. 2021 Feb 8;16(2):e0246335. doi: 10.1371/journal.pone.0246335 (PMC7870052; doi:10.1371/journal.pone.0246335)
Supplement: S1 Table — (DOCX) [file pone.0246335.s001.docx]

**S1 Table. Length-weight conversion formula to estimate weight of fish from their total lengths, and *a* and *b* coefficients for each studied species.**

W=a L ^b^, being W the weight (g) of the fish and L its total length (cm). *a* and *b* are species specific and have being extracted from local studies when available (Morey et al. 2003) and Fishbase (Froese & Pauly 2017).

| **Family** | **Species** | ***a*** | ***b*** |
| --- | --- | --- | --- |
| Myliobatidae | *Myliobatis aquila* | 0.00389 | 3.09 |
| Clupeidae | *Sarda sarda* | 0.00813 | 3.05 |
| Engraulidae | *Engraulis encrasicolus* | 0.0048 | 3.0706 |
| Muraenidae | *Muraena helena* | 0.0006 | 3.2736 |
| Belonidae | *Belone belone* | 0.00093 | 3.05 |
| Phycidae | *Phycis phycis* | 0.0045 | 3.2681 |
| Serranidae | *Anthias anthias* | 0.01047 | 2.94 |
|  | *Epinephelus costae* | 0.0134 | 2.9671 |
|  | *Epinephelus marginatus* | 0.0104 | 3.121 |
|  | *Epinephelus caninus* | NA. Values taken from *E. costae* | |
|  | *Mycteroperca rubra* | 0.01072 | 3.01 |
|  | *Serranus atricauda* | 0.00813 | 3.09 |
|  | *Serranus cabrilla* | 0.0092 | 3.0658 |
|  | *Serranus scriba* | 0.0104 | 3.1103 |
| Moronidae | *Dicentrarchus labrax* | 0.0051 | 3.1589 |
| Apogonidae | *Apogon imberbis* | 0.0093 | 3.2021 |
| Carangidae | *Seriola dumerili* | 0.0273 | 2.7438 |
|  | *Trachurus spp.* | 0.00891 | 2.96 * |
|  | *Pseudocaranx dentex* | 0.01413 | 2.96 |
| Scombridae | *Sarpa salpa* | 0.0323 | 2.7004 |
|  | *Euthynnus aletteratus* | 0.01 | 3.05 |
| Coryphaenidae | *Coryphaena hippurus* | 0.0138 | 2.87 |
| Haemulidae | *Pomadasys incisus* | 0.01148 | 2.90 |
|  | *Parapristipoma octolineatum* | 0.01288 | 3.01 |
| Sciaenidae | *Sciaena umbra* | 0.0053 | 3.2542 |
| Mullidae | *Mullus surmuletus* | 0.0073 | 3.1685 |
| Sparidae | *Boops boops* | 0.0119 | 2.8554 |
|  | *Dentex dentex* | 0.0113 | 3.0349 |
|  | *Diplodus annularis* | 0.0115 | 3.1668 |
|  | *Diplodus cervinus* | 0.01202 | 3.08 |
|  | *Diplodus puntazzo* | 0.026 | 2.8188 |
|  | *Diplodus sargus* | 0.0114 | 3.1317 |
|  | *Diplodus vulgaris* | 0.0149 | 3.0058 |
|  | *Oblada melanura* | 0.01202 | 3.02 |
|  | *Pagrus pagrus* | 0.0282 | 2.8003 |
|  | *Pagrus auriga* | NA. Values taken from *P. pagrus* | |
|  | *Sardina pilchardus* | 0.0075 | 2.9577 |
|  | *Sparus aurata* | 0.0053 | 3.2393 |
|  | *Spondyliosoma cantharus* | 0.0158 | 2.9957 |
|  | *Spicara smaris* | 0.0113 | 2.8696 |
|  | *Spicara maena* | 0.0113 | 3.0649 |
| Pomacentridae | *Chromis chromis* | 0.0189 | 2.9271 |
| Labridae | *Coris julis* | 0.007 | 3.0462 |
|  | *Labrus merula* | 0.0076 | 3.1862 |
|  | *Labrus viridis* | 0.0058 | 3.2216 |
|  | *Symphodus dordeleini* | 0.0083 | 3.155 |
|  | *Symphodus mediterraneus* | 0.0123 | 3.0653 |
|  | *Symphodus melanocercus* | NA. Values taken from *S. rostratus* | |
|  | *Symphodus ocellatus* | 0.0131 | 2.9664 |
|  | *Symphodus roissali* | 0.01148 | 2.99 |
|  | *Symphodus cinereus* | 0.0075 | 3.2514 |
|  | *Symphodus rostratus* | 0.0069 | 3.2169 |
|  | *Symphodus tinca* | 0.0184 | 2.8757 |
|  | *Thalasoma pavo* | 0.00977 | 3.05 |
| Sphyraenidae | *Sphyraena viridensis* | 0.0016 | 3.1831 |
| Mugilidae | *Mugilidae spp.* | 0.00977 | 2.96** |
| Scorpaenidae | *Scorpaena maderensis* | 0.0164 | 3.0354 |
|  | *Scorpaena scrofa* | 0.022 | 2.9418 |
|  | *Scorpaena porcus* | 0.0183 | 3.0202 |
|  | *Scorpaena notata* | 0.01689 | 3.0384 |
| Atherinidae | *Atherina sp.* | 0.00562 | 3.07 *** |
| Molidae | *Mola mola* | 0.02455 | 3.02 |

* Taken from *Trachurus mediterraneus*

**Taken from *Liza aurata*

***Taken from *Atherina hepsetus*
